# Prognostic Significance of Systematic Lymphadenectomy in Patients With Optimally Debulked Advanced Ovarian Cancer: A Meta-Analysis

**DOI:** 10.3389/fonc.2020.00086

**Published:** 2020-02-11

**Authors:** Yizi Wang, Fang Ren, Zixuan Song, Xiaoying Wang, Chiyuan Zhang, Ling Ouyang

**Affiliations:** Department of the Obstetrics and Gynecology, Shengjing Hospital of China Medical University, Shenyang, China

**Keywords:** advanced ovarian cancer, optimal debulking surgery, residual tumor, systematic lymphadenectomy, meta-analysis

## Abstract

**Background:** The effect of systematic lymphadenectomy (SL) on survival in patients with optimally debulked advanced ovarian cancer remains unclear. We evaluated the therapeutic value of SL in advanced ovarian cancer patients who underwent primary optimal debulking surgery.

**Methods:** A meta-analysis was carried out using articles retrieved from the PubMed, Embase, and Cochrane databases. Overall survival (OS) and progression-free survival (PFS) were compared between patients who underwent SL and those who underwent unsystematic lymphadenectomy (USL).

**Results:** Seven studies that included 2,425 patients with advanced ovarian cancer were included in the meta-analysis. The overall analyses indicated significantly improved OS [hazard ratio (HR) = 0.64, 95% confidence interval (CI): 0.49–0.84, *P* < 0.01] but not PFS (HR = 0.89, 95% CI: 0.69–1.15, *P* = 0.38) in patients who underwent SL compared to those who underwent USL. Subgroup analyses based on study type, study quality, total numbers of patients, and International Federation of Gynecology and Obstetrics (FIGO) stage provided similar results. However, subgroup analysis of patients with no residual tumor revealed that SL was not associated with improved OS (HR = 0.81, 95% CI: 0.66–1.00, *P* = 0.05) or PFS (HR = 1.09, 95% CI: 0.91–1.30, *P* = 0.33).

**Conclusions:** In patients with optimally debulked advanced ovarian cancer, SL may improve OS but not PFS. However, SL does not provide a survival advantage when macroscopically complete resection of all visible tumors is achieved.

## Introduction

Ovarian cancer is the second most common cancer (1). In 2018, there were 295,414 new cases of ovarian cancer and 184,799 deaths due to ovarian cancer worldwide (2). Primary debulking surgery with the goal of macroscopically complete resection of all visible tumors followed by platinum/taxane-based chemotherapy is the primary treatment for advanced ovarian cancer (3). Optimal debulking surgery is defined by a resulting residual tumors <1 cm at the largest diameter (4). Some studies have reported that complete and optimal debulking surgery can improve survival outcomes (5, 6). Lymphatic spread is commonly observed in both early and advanced ovarian cancer (7), and retroperitoneal lymph node metastasis has been reported to be related to a poor prognosis (8). However, it is unclear whether systematic lymphadenectomy (SL) can improve survival outcomes, especially in patients with optimally debulked advanced ovarian cancer (9).

Only a few meta-analyses have compared systematic lymphadenectomy (SL) with unsystematic lymphadenectomy (USL) in a population of ovarian cancer patients with all International Federation of Gynecology and Obstetrics (FIGO) stages, and different conclusions have been reached (10, 11). In addition, several observational studies have compared overall survival (OS) or progression-free survival (PFS) in patients with completely or optimally debulked advanced ovarian cancer, and inconsistent results were obtained (12, 13).

The results of a phase 3, multicenter, randomized trial of lymphadenectomy in patients with advanced ovarian cancer were recently published, and the study found that SL did not improve the OS or PFS relative to not performing lymphadenectomy in patients with completely resected advanced ovarian cancer (14).

Thus, the purpose of this study was to perform a meta-analysis to determine the impact of SL on survival in patients with optimally debulked advanced ovarian cancer.

## Methods

### Search Strategy and Data Sources

This meta-analysis was conducted according to the PRISMA (Preferred Reporting Items for Systematic Reviews and Meta-Analyses) guidelines (http://www.prisma-statement.org/). The checklist in accordance with PRISMA is shown in Supplementary Table 1. The PubMed, Embase, and Cochrane databases were systematically searched through August 15, 2019. The following search terms were used: “lymph node excision,” “excision, lymph node,” “lymphadenectomy,” “lymphadenectomies,” “lymph node dissection,” “dissection, lymph node,” “lymph node dissections,” “node dissection, lymph” and “ovarian neoplasm,” “ovary neoplasm,” “ovary cancer,” “ovarian cancer,” “cancer of ovary.” There were no restrictions with regard to language. The references of the selected studies were also examined to identify additional relevant studies.

### Study Selection

Based on the PICOS (population, intervention, comparison, outcomes and study design) guidelines, studies were selected according to the following inclusion criteria: (1) Population: patients with advanced ovarian cancer with a FIGO stage of IIB through IV; (2) Intervention: optimal debulking surgery (residual tumor < 1 cm) was the primary treatment; (3) Comparison: patients received SL vs. USL, including no lymphadenectomy or resection of bulky nodes only (adjuvant therapy administered to both groups); (4) Outcomes: OS and PFS compared between SL group and USL group; and (5) Study design: comparative studies including randomized control trials (RCTs) or observational studies.

The exclusion criteria were as follows: (1) patients with early ovarian cancer or ovarian borderline malignancies; and (2) patients who underwent neoadjuvant chemotherapy or patients with residual tumor(s) > 1 cm.

### Data Extraction and Study Quality Assessment

Two reviewers (YW and FR) reviewed and assessed the included studies. Data extraction was conducted independently, and the following information was extracted from the included studies: first author, publication year, country in which study was conducted, study type, setting, study period, follow-up time, number of advanced ovarian cancer patients enrolled, FIGO stage, residual tumor, and survival data (OS and PFS). The quality of the RCTs was assessed using the Jadad scale (15), and the quality of the observation studies was assessed using the Newcastle-Ottawa Scale (NOS) (16). All disagreements were resolved through discussion.

### Statistical Analysis

Hazard ratios (HRs) were used to assess the primary endpoints (time-to-event outcomes). If studies did not provide the HR directly, an estimated HR was calculated from Kaplan-Meier curves based on the method developed by Tierney (17). All analyses were carried out using Stata software, version 12.0 (2011; Stata Corp., College Station, TX, USA). HRs are presented with 95% confidence intervals (CIs). A two-tailed value of *P* < 0.05 was considered to indicate statistical significance. Heterogeneity among studies was measured by Cochran's Q test (reported with a χ^2^ value and *P*-value) and *I*^2^ statistics (18, 19). The low, moderate, and high levels of heterogeneity were indicated by *I*^2^ values of 25, 50, and 75%, respectively (20). Study heterogeneity was examined, and a random-effects model was used in all analyses. Sensitivity analyses were conducted to evaluate the robustness of the results (21). Subgroup analyses were performed to detect sources of heterogeneity and to further assess the impact of SL. Subgroup analyses were based on study type, study quality, total patients, FIGO stage, and residual tumor. Funnel plots were used to assess publication bias (22), and Begg's and Egger's regression were used to test for funnel plot asymmetry (23, 24).

## Results

### Study Selection

**Figure 1:**
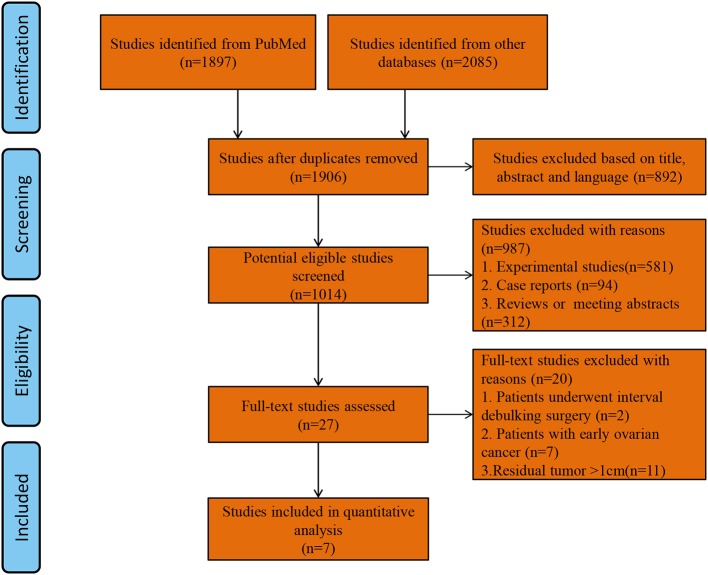
Flow diagram of study selection.

A total of 1,906 studies were identified by the search strategy. After eliminating duplicate studies and screening articles by title and abstract, the full texts of 27 studies were reviewed. Finally, seven studies with a total of 2,425 patients (SL group = 1,378, USL group = 1,047) who met the inclusion criteria were included in the analysis (9, 12–14, 25–27). A flow diagram of study selection is shown in Figure 1. Of the seven studies, two were RCTs and 5 were observational studies. The two RCTs had total Jadad scores ≥ 3 and thus were considered to be of high quality. The four observational studies had NOS scores ≥ 7 and thus were considered to be of high quality. The main characteristics of the study populations in the included studies and the quality of the included studies are presented in Table 1.

**Table 1:** Baseline characteristics of the included studies.

**Study**	**Country**	**Study period**	**Study type**	**Setting**	**Stage**	**Residual tumor**	**Follow-up (median/mean months)**	**Total patients**	**SL**	**USL**	**Studies quality**
Harter et al. (14)	Germany	2008–2012	RCT	Multi	IIB-IV	0	72	647	323	324	7^*^
Eoh et al. (12)	Korea	2006–2015	Observational	Single	IIIC-IV	<1 cm	NA	158	96	62	5^#^
Paik et al. (26)	Korea	2002–2013	Observational	Single	III-IV	<1 cm	48	159	102	57	8^#^
Sakai et al. (13)	Japan	1986–2009	Observational	Single	III-IV	0/ <1 cm	49.6	180	87	93	7^#^
du Bois et al. (25)	Germany	1995–2002	RCT	Multi	IIB-IV	0	56	996	658	338	6^*^
Aletti et al. (27)	USA	1994–1998	Observational	Single	IIIC-IV	<1 cm	36	187	61	126	8^#^
Isonishi et al. (29)	Japan	1992–2000	Observational	Single	IIIC-IV	<1 cm	24	98	51	47	8^#^

*SL, systematic lymphadenectomy; USL, unsystematic lymphadenectomy; RCT, randomized controlled trial; NA, not applicable*.

* *Jadad scale was used to assess the quality of the randomized clinical trials*.

# *The Newcastle-Ottawa Scale was used to assess the quality of the observational studies*.

### Overall Meta-Analyses of OS and PFS

**Figure 2:**
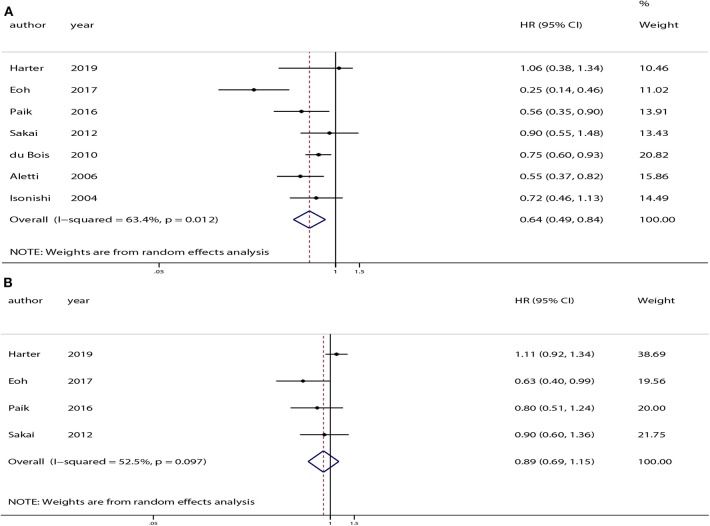
Overall analyses of systematic lymphadenectomy vs. unsystematic lymphadenectomy for advanced ovarian cancer patients. **(A)** Overall survival (OS). **(B)** Progression-free survival (PFS).

All of the studies provided OS data. The pooled analysis indicated that compared with USL, SL significantly improved OS (HR = 0.64, 95% CI: 0.49–0.84, *P* < 0.01). PFS was only available in four of the studies (12–14, 26). The pooled analysis indicated no significant difference in PFS between the SL and USL groups (HR = 0.89, 95% CI: 0.69–1.15, *P* = 0.38). The overall meta-analysis results of OS and PFS are shown in Figure 2.

**Figure 3:**
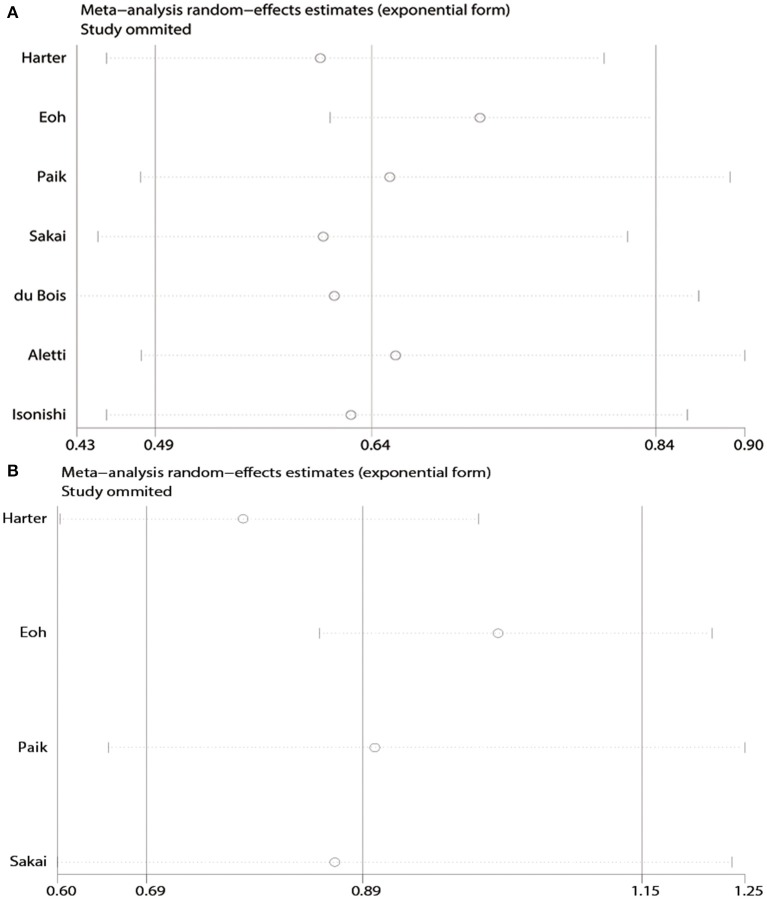
Sensitivity analyses of the pooled meta-analysis. **(A)** Overall survival (OS). **(B)** Progression-free survival (PFS).

Moderate heterogeneity was observed among the studies with respect to OS (χ^2^ = 16.37, *P* = 0.01, *I*^2^ = 63.4%) and PFS (χ^2^ = 6.32, *P* = 0.10, *I*^2^ = 52.5%). Sensitivity analyses conducted by excluding studies one-by-one found that none of the individual studies affected the pooled HRs of OS or PFS (Figure 3).

**Figure 4:**
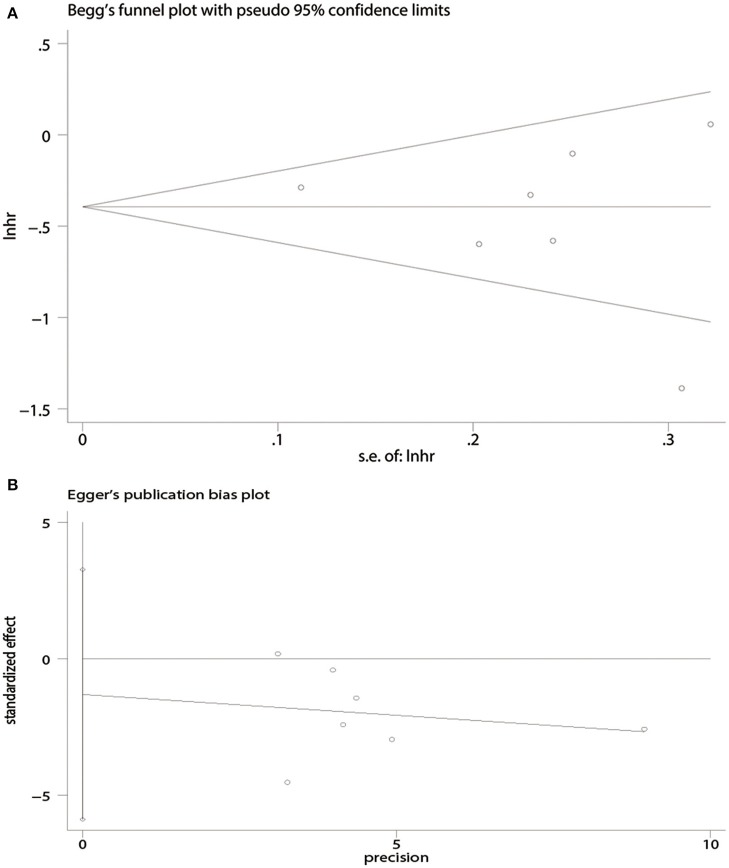
Publication bias for overall survival (OS) analysis. **(A)** Begg's test. **(B)** Egger's test.

**Figure 5:**
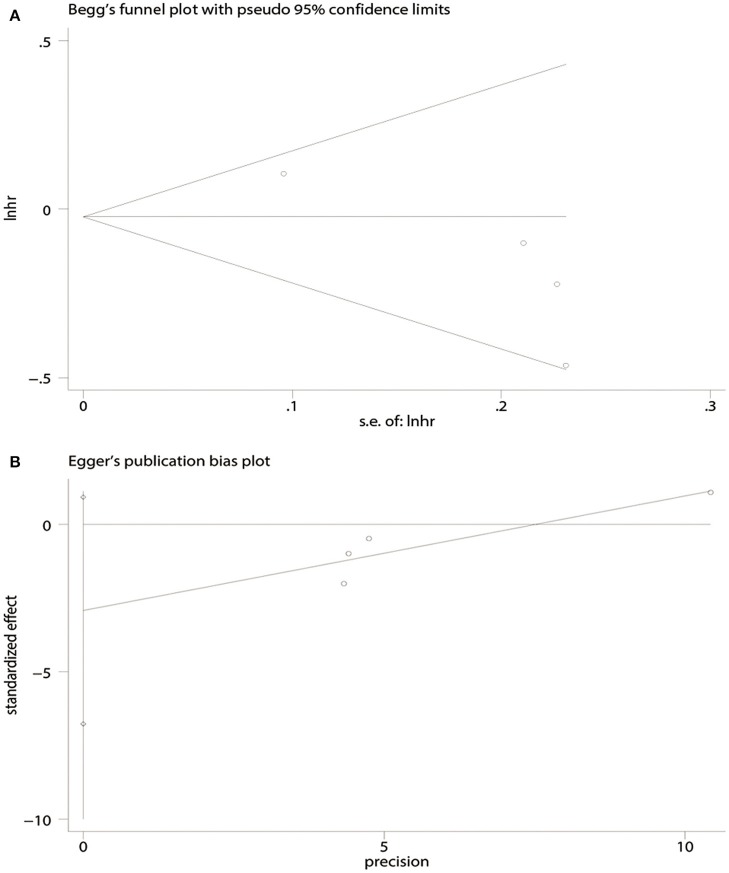
Publication bias in terms of progression-free survival (PFS) analysis. **(A)** Begg's test. **(B)** Egger's test.

There was no evidence of publication bias with respect to OS [Begg's test, *P* = 1.00 (Figure 4A); Egger's test, *P* = 0.50 (Figure 4B)] or PFS [Begg's test, *P* = 0.09 (Figure 5A); Egger's test, *P* = 0.08 (Figure 5B)].

### Subgroup Analyses

#### Subgroup Analyses Based on Study Type, Study Quality, and Total Patients

Subgroup analyses of OS and PFS were conducted based on study type, study quality, and total patients. Compared to USL, SL was associated with a significant improvement in OS in the RCTs (HR = 0.78, 95% CI: 0.63–0.97, *P* = 0.03) and observational studies (HR = 0.56, 95% CI: 0.39–0.81, *P* < 0.01). However, SL was not associated with improved PFS in the RCTs (HR = 1.11, 95% CI: 0.92–1.34, *P* = 0.28) or observational studies (HR = 0.78, 95% CI: 0.60–1.00, *P* = 0.05). Subgroup analysis by study quality found that SL was associated with increased OS in high quality studies, but no increase in PFS was observed. Subgroup analyses based on total number of patients (>500 or <500 patients) indicated that SL significantly improved OS but not PFS (Table 2).

**Table 2:** Subgroup analyses of systematic lymphadenectomy and survival outcomes in patients with advanced ovarian cancer.

**Characteristics**	**OS**				**PFS**			
	**Study number**	**HR (95%CI)**	***P*-value**	**Heterogeneity**	**Study number**	**HR (95%CI)**	***P*-value**	**Heterogeneity**
**Study type**	7	0.64 (0.49, 0.84)	<0.01	63.4%	4	0.89 (0.69, 1.15)	0.38	52.5%
RCT	2	0.78 (0.63, 0.97)	0.03	3.2%	1	1.11 (0.92, 1.34)	0.28	0.0%
Observational studies	5	0.56 (0.39, 0.81)	<0.01	65.5%	3	0.78 (0.60, 1.00)	0.05	0.0%
**Total patients**								
≥500	2	0.78 (0.63, 0.97)	0.03	3.2%	1	1.11 (0.92, 1.34)	0.28	0.0%
<500	5	0.56 (0.39, 0.81)	<0.01	65.5%	3	0.78 (0.60, 1.00)	0.05	0.0%
**Quality of studies**								
High	6	0.72 (0.61, 0.84)	<0.01	4.5%	3	1.02 (0.85, 1.22)	0.85	10.5%
Low	1	0.25 (0.14, 0.46)	<0.01	0.0%	1	0.63 (0.40, 0.99)	0.05	0.0%
**FIGO stage**								
IIB-IV	2	0.78 (0.63, 0.97)	0.03	3.2%	1	1.11 (0.92, 1.34)	0.28	0.0%
III-IV	5	0.56 (0.39, 0.81)	<0.01	65.5%	3	0.78 (0.60, 1.00)	0.05	0.0%
**Residual tumor**								
No	3	0.81 (0.66, 1.00)	0.05	2.9%	2	1.09 (0.91, 1.30)	0.33	0.0%
**<1 cm**	4	0.50 (0.34, 0.74)	<0.01	61.9%	2	0.71 (0.52, 0.98)	0.04	0.0%

*OS, overall survival; PFS, progression-free survival; HR, Hazard Ratio; RCT, randomized controlled trial*.

#### Subgroup Analyses Based on FIGO Stage and Absence of Residual Tumor

**Figure 6:**
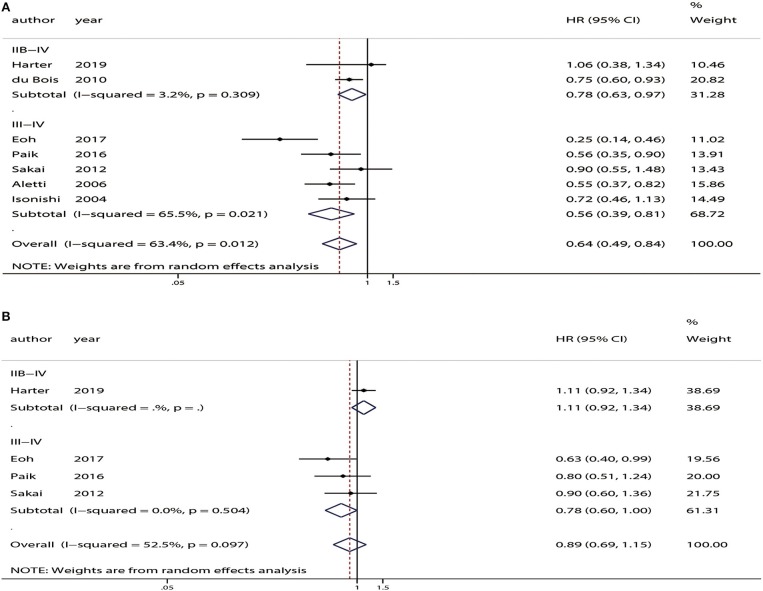
Subgroup analyses based on the International Federation of Gynecology and Obstetrics (FIGO) stage between patients who underwent systematic lymphadenectomy and those who underwent unsystematic lymphadenectomy. **(A)** Overall survival (OS). **(B)** Progression-free survival (PFS).

Subgroup analyses of OS and PFS based on FIGO stage found that SL significantly improved OS in patients with FIGO stage IIB-IV disease (HR = 0.78, 95% CI: 0.63–0.98, *P* = 0.03) and stage III-IV disease (HR = 0.56, 95% CI: 0.39–0.81, *P* < 0.01). However, no improvement in PFS was observed for any FIGO stage (Figure 6).

**Figure 7:**
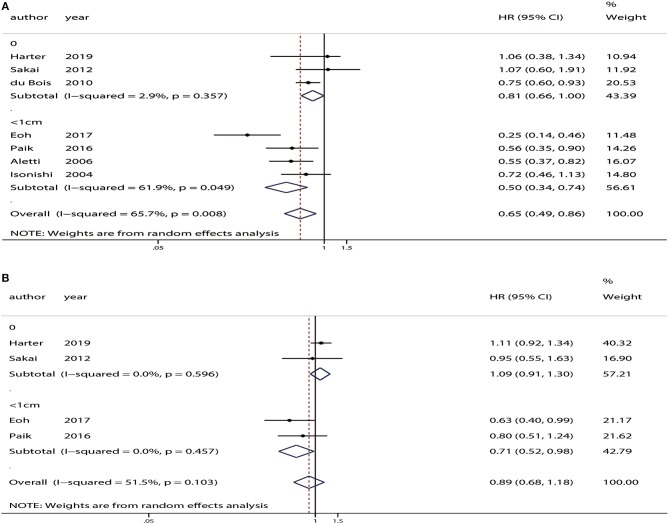
Subgroup analyses based on residual tumor status in advanced ovarian cancer patients who underwent systematic lymphadenectomy vs. those who underwent unsystematic lymphadenectomy. **(A)** Overall survival (OS). **(B)** Progression-free survival (PFS).

Three studies included advanced ovarian cancer patients with no residual tumor (13, 14, 25). Analysis of these patients indicated that there was no difference in OS (HR = 0.81, 95% CI: 0.66–1.00, *P* = 0.05) or PFS (HR = 1.09, 95% CI: 0.91–1.30, *P* = 0.33) between patients who received SL and those who received USL (Figure 7).

## Discussion

Approximately 1.3% of women will develop ovarian cancer throughout their lifetime, and most patients are advanced by the time it is diagnosed (28), making ovarian cancer the leading cause of death from gynecologic cancer (4). Optimal debulking (residual tumor < 1 cm) is an important independent prognostic factor for survival in advanced ovarian cancer patients (29, 30). However, the indications for performing SL have been inconclusive due to the constant updates of the NCCN guidelines.

Over the past decade, several studies have compared the outcomes of advanced ovarian cancer patients who received SL or USL, and the results have been inconsistent. In 2014, Gao et al. (10) and in 2016, Zhou et al. (11) performed meta-analyses evaluating the outcomes of SL and USL. However, these studies analyzed ovarian cancer patients with all FIGO stages and did not take residual tumor status into account. With the recent publication of the RCT results comparing the performance of SL or not for advanced ovarian cancer patients, the issue of performing SL in advanced ovarian cancer has received the attention of gynecologists once again. In addition, it is time to evaluate the survival outcomes associated with SL.

Our meta-analysis included seven studies with 2,425 patients with advanced ovarian cancer. The results showed that SL was associated with an improvement in OS in optimally debulked advanced ovarian cancer patients but did not improve PFS. Subgroup analyses based on study type, study quality, and total patients also indicated that SL improved OS but not PFS. Subgroup analysis by FIGO stage also indicated that SL improved OS but not PFS.

Different factors may affect OS and PFS. Site of recurrence and number of nodules (31), sensitivity to chemotherapy and treatment for recurrence (32) have been shown to be associated with postrecurrence survival. Gallotta et al. (33) reported that metastatic mesenteric lymph nodes were found in almost half of the cases with detectable mesenteric lymph nodes, which induced recurrences of ovarian cancer more frequently. In addition, hepatoceliac lymph nodes (HCLNs) should be resected for assessment of HCLN involvement, as they have been associated with worse PFS (34). Moreover, tumor response to primary treatments (35) and disease-free interval (36) are independent prognostic factors for OS. In addition, patients who undergo USL might have more lymphatic metastasis, which could be more chemo-resistant (37). All of the aforementioned factors may contribute to poorer postrecurrence survival in patients who undergo USL.

Macroscopically complete resection followed by combination chemotherapy provides the best outcomes for advanced ovarian cancer patients (38). Results from a high-quality RCT (14) reported that SL did not improve the OS or PFS of advanced ovarian cancer patients with macroscopically complete resection. Thus, we conducted a subgroup analysis based on residual tumor status that included three studies and found no significant difference in OS or PFS between SL and USL patients. There are some possible explanations for these findings. Residual tumors might play a dominant role in the prognosis of advanced ovarian cancer patients, and if macroscopically complete resection is performed, SL might not be necessary. Additionally, many studies have reported a higher frequency of intraoperative or postoperative complications in patients who received lymphadenectomy, including intraoperative hemorrhage, higher rates of blood transfusions, and lymphocele (14, 39–41). Hence, if macroscopically complete resection had been achieved in advanced ovarian cancer patients, SL might not be performed. However, the number of studies included in our analysis was limited, and the results should be verified by more RCTs with large numbers of patients.

To the best of our knowledge, this was the first meta-analysis to explore the association between SL and OS and PFS in advanced ovarian cancer patients. The inclusion and exclusion criteria were based on PICOS criteria, and the Jadad scale and NOS criteria were used to evaluate the quality of the RCTs and observational studies, respectively; the majority of the included studies were of high quality.

However, there are some limitations to our meta-analysis. First, the heterogeneity of the included studies was significant. Because most of the studies were retrospective and conducted in single centers, they might contain selection, information, and confounding biases. The criteria for candidate selection for SL may differ between centers and surgeons. Lymphadenectomy may be performed in fitter or younger patients, rather than patients with a poor health status, so the criteria may not depend on the disease characteristics only. Thus, patients with more advanced disease might undergo USL rather than SL, and this bias cannot be avoided in retrospective analyses (14). Second, the group of patients who underwent USL included those who received no lymphadenectomy or who underwent resection of bulky nodes only. Patients with bulky nodes might have poorer tumor characteristics, which might lead to poorer outcomes (41). Third, many factors could influence the prognosis and therapeutic approach of ovarian cancer, such as histological types and biological manifestations, but we could not combine the results due to the limited number of studies. Fourth, the number of studies included was relatively small, especially studies that included PFS data. Finally, in most studies included, OS was described as death from any cause, but disease-specific OS is most relevant. Moreover, PFS did not differ between the SL and USL groups. Hence, we could not conclude definitively that SL was associated with better disease-specific OS.

## Conclusion

SL may improve OS but not PFS in patients with optimally debulked advanced ovarian cancer. However, if macroscopically complete resection was performed, SL offers no improvement in OS or PFS compared to USL. Further studies based on large, well-designed, high-quality RCTs are needed to confirm our findings.

## Data Availability Statement

All datasets generated for this study are included in the article/Supplementary Material.

## Author Contributions

LO and YW designed the study idea and the study methodology. YW and FR conducted the research and analyzed data. ZS, CZ, and XW participated in the coordination of the study and provided specific support in quantitative data analysis. YW wrote the manuscript. All authors read and approved the version of the manuscript.

### Conflict of Interest

The authors declare that the research was conducted in the absence of any commercial or financial relationships that could be construed as a potential conflict of interest.
